# Noninvasive Method for Monitoring *Pneumocystis carinii* Pneumonia

**DOI:** 10.3201/eid0912.030270

**Published:** 2003-12

**Authors:** Michael J. Linke, Sandy Rebholz, Margaret Collins, Reiko Tanaka, Melanie T. Cushion

**Affiliations:** * Veterans Affairs Medical Center, Cincinnati, Ohio, USA; † University of Cincinnati College of Medicine, Cincinnati, Ohio, USA

**Keywords:** pneumonia, *Pneumocystis carinii*, fungal, polymerase chain reaction, lung diseases, fungal, bronchoalveolar lavage

## Abstract

The progression of *Pneumocystis carinii* pneumonia was temporally monitored and quantified by real-time polymerase chain reaction of *P. carinii*–specific DNA in oral swabs and lung homogenates from infected rats. DNA levels correlated with the number of *P. carinii* organisms in the rats’ lungs, as enumerated by microscopic methods. This report is the first of a noninvasive, antemortem method that can be used to monitor infection in a host over time.

*Pneumocystis* pneumonia remains a leading opportunistic infection associated with AIDS patients, even in the era of highly active antiretroviral therapy (*1*). In developing countries, the incidence of infection has increased dramatically, with mortality rates ranging from 20% to 80% (*2*). An important limitation in its clinical management has been the inability to evaluate therapeutic response or to temporally measure the organism numbers because of the absence of an in vitro culture system. Our laboratory recently showed that the presence of *Pneumocystis carinii*–specific amplicons obtained from swabs of the oral cavities of nonimmunocompromised adult rats *(Rattus norvegicus)* was predictive of the development of *P. carinii* pneumonia after corticosteroid-induced immunosuppression (*3*). In the present study, we applied the oral swab technique in combination with quantification of organism-specific DNA using real-time polymerase chain reaction (PCR) to monitor the progression of infection in the rat model.

## The Study

Thirty-two male Long Evans rats (140–160 g) known to harbor *P. carinii* were obtained from Room 004 at the Cincinnati Veterinary Medical Unit (*4*). All rats produced *P. carinii* amplicons from initial oral swab samples taken before immunosuppression. After sampling, 8 of the 32 rats were euthanized and their lungs were removed and processed as described below. The remaining 24 rats were removed from the room and individually caged under barrier conditions, as described previously (*3*), to prevent transmission of infection that might occur between cage mates or from the environment. Barrier conditions consisted of the following: microisolator tops for each shoebox cage, which was then housed within a BioBubble (The Colorado Clean Room Company, Fort Collins, CO); autoclaved water, into which a sterile solution of cephadrine (Velosef; E.R. Squibb and Sons, Inc., Princeton, NJ) was injected for a final concentration of 0.200 mg/mL; autoclaved cages, bedding, and tops; and irradiated Lab Chow (Tekmar Irradiated Lab Chow, Harlan Industries, Indianapolis, IN). To provoke *P. carinii* pneumonia, 4 mg/kg of methylprednisolone acetate (Depo Medrol; The Upjohn Co., Kalamazoo, MI) was administered to the rats weekly for 10 weeks. At 4 and 7 weeks, swab samples were obtained from groups of eight rats; the rats were then euthanized. Their lungs were removed for quantification by microscopic enumeration of organism nuclei expressed as log nuclei/mL (*5*) and real-time PCR analysis under aseptic conditions. Six rats survived the 10 weeks of immunosuppression and were processed in an identical manner.

DNA was extracted from the oral swabs (OS) and lung homogenate (LH), as previously described (*4*). LH DNA was evaluated by spectrophotometric analysis at 260 and 280 nm. RC primers directed to a region of the mitochondrial large subunit rRNA (mtLSU) were used for amplification of *P. carinii*–specific DNA (*6*).

Real-time PCR was performed and results were analyzed on the iCycler iQ Real-Time PCR Detection System (BioRad Laboratories, Hercules, CA) under conditions of rapid melting at 95°C, annealing for 5 s at 55°C, and collection at 76° C for 10 s with 40 cycles of amplification. Five microliters of a 1/5 dilution of OS DNA or 2.5 ng of LH DNA were used in the reactions. Taq DNA (1.25 U) polymerase (Promega, Madison, WI) was used in the real-time PCR with a concentration of 2.5 mM MgCl_2_ in 25-μL reactions. To monitor the accumulation of the products, 0.4 μL of 1/1,000 dilution of concentrated SYBR Green (Molecular Probes, Eugene, OR) was included in the reactions. All reactions were performed in triplicate. The mtLSU product was cloned into the TOPO-TA PCR cloning vector (InVitrogen, Carlsbad CA) (mtLSU-T-TA), quantified by spectrophotometry, and used to generate a standard curve. The cloned PCR product, ranging from 0.0005 pg to 0.5 pg per reaction, was used as a template; the threshold cycles (C_T_s) of these reactions were then plotted against the log amount of plasmid per reaction in picograms.

*P. carinii* DNA in the LH and OS samples was quantified by linear regression analysis of the C_T_s relative to the standard curve (*3*). The concentration of *P. carinii* DNA in the LH and OS samples, determined from the standard curve in picograms, was converted to copies per milliliter by multiplying by the dilution factor based on the original concentration of DNA. The LH copies were log transformed and expressed as log copies per milliliter. The specificity of the reactions was verified by analysis of the product-melting curves and by gel electrophoresis. All products were of the expected size (137 bp) and produced a single peak with a T_m_ of approximately 78°C.

Microscopic enumeration of nuclei of the lung homogenates was compared to real-time PCR lung homogenate results by using Tukey-Kramer Multiple Comparisons post-test to assess significance (InStat version 3; GraphPad Software, Inc., San Diego, CA). Pre- and postimmunosuppression OS samples were analyzed with the Mann-Whitney test (InStat v. 3). Spearman Rank Correlation was used to evaluate the correlation between microscopic enumeration and the real-time PCR output (Instat v.3).

To ensure accurate and reproducible results, the efficiency of the real-time PCR with the RC primer set was evaluated for each type of sample used in this study: mtLSU/T-TA, LH DNA, and OS DNA (Table 1). The exponential amplification and efficiency of the reactions were determined by evaluating the slope of the curve generated by plotting the log of known concentrations of template DNA vs. their C_T_s (*7*). The RC primer set demonstrated acceptable levels of exponential amplification and efficiency with all three templates.

**Table 1:** Efficiencies of the real-time PCR reactions^a^

Template	Range	r^2^	Slope	Amplification	Efficiency	
mtLSU	0.5 to 0.0005 pgs/rx	0.999	-3.220	2.043	1.043	
LH	12.5 to 1.25 x 10-5 ngs/rx	0.966	-3.570	1.906	0.906	
OS	Undiluted to 1:8 dilution	0.973	-3.328	1.997	0.997	

^a^PCR, polymerase chain reaction; mtLSU, mitochondrial large ribosomal subunit RNA; LH, rat lung homogenate; OS, oral swab *P. carinii*–specific DNA; rx, reaction.

The organism numbers in lung tissue, quantified by microscopic enumeration, increased from log 4.69 after 4 weeks of immunosuppression to log 9.35 after 10 weeks of immunosuppression (Figure, A.). No organisms were detected in the lungs of the eight rats euthanized before the study began (level of sensitivity = ~10,000 nuclei per lung). The amount of *P. carinii*–specific DNA quantified by real-time PCR in the LH samples increased substantially from 0 to 7 weeks, with similar levels after 7 and 10 weeks of immunosuppression (Figure, B). Only one of eight rats euthanized at the initiation of the experiment produced quantifiable copies of *P. carinii*–specific DNA, with a level similar to those after 4 weeks of immunosuppression (data not shown). In every case, the postimmunosuppression OS taken from the rats at 4, 7, and 10 weeks had significantly more *P. carinii*–specific DNA than the preimmunosuppression OS taken at the initiation of the study (Figure, C). The amount of *P. carinii*–specific DNA in the OS samples also increased over time (Figure, C). No significant correlation was found between the amount of *P. carinii* DNA detected in the preimmunosuppression OS samples and the amount in the postimmunosuppression OS samples, the lung homogenates, or nuclei number, suggesting that the rats had equivalent but low levels of organisms at the initiation of the study.

**Figure:**
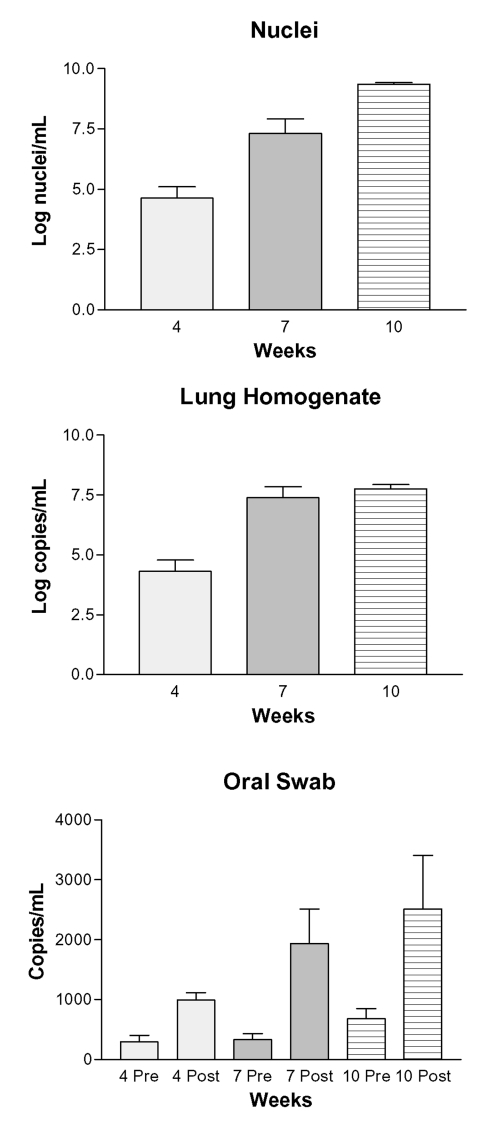
Progression of *Pneumocystis carinii* pneumonia measured by enumeration of organisms and real-time PCR of DNA extracted from lung homogenates and oral swabs. Panel A: Log *P. carinii* nuclei per mL of homogenized rat lung assessed by microscopic enumeration of lung homogenates; 4 wk vs. 7 wk, p < 0.001; 4 wk vs. 10 wk, p < 0.001; 7 wk vs. 10 wk, p < 0.001. Panel B: Log-transformed copies of *P. carinii-*specific DNA (mtLSU) per mL of lung homogenate; 4 wk vs. 7 wk, p < 0.001; 4 wk vs. 10 wk, p < 0.001; 7 wk vs. 10 wk, p > 0.05. Panel C: Copies of *P. carinii*-specific DNA/mL in oral swabs taken between immunosuppression (4, 7, and 10 wk preimmunosuppression) and at the time of euthanasia (4, 7, and 10 wk postimmunosuppression); pre- and post-4 wk, p = 0.0006; pre- and post-7 wk, p = 0.0037; pre- and post-10 wk, p = 0.0221. The ranges in value for the preimmunosuppression oral swabs at 4, 7, and 10 wk were, respectively, 165–487 copies/mL; 98–816 copies/mL; and 380–1,667 copies/mL. Ranges for the postimmunosuppression copies/mL after 4, 7, and 10 wk were 342–1,254; 517–5,256; and 637–6,279. (Number of rats in the 4- and 7- wk postimmunosuppression group = 8; number of rats in the 10-wk group = 6.)

To determine the relationship between quantitation of *P. carinii* by real-time PCR and by microscopic enumeration, results were analyzed by Spearman Rank Correlation (Table 2). A significant correlation was found between both the amount of *P. carinii* DNA detected in the postimmunosuppression OS samples and in the LH versus the number of *P. carinii* nuclei. A significant correlation was also detected between the real-time PCR quantitation of *P. carinii* DNA in the OS and the LH.

**Table 2:** Comparisons of *Pneumocystis carinii*–specific DNA levels in pre- and postimmunosuppression samples, lung homogenates, and organism numbers in lung homogenates assessed by microscopic enumeration^a^

Groups^b^	No. points	Spearman r	95% Confidence interval	p value	Significant
LH Pc DNA vs. post-OS Pc DNA	22	0.5576	0.1648 to 0.7978	0.0070	Yes
LH Pc DNA vs. Pc nuclei	22	0.9035	0.7731 to 0.9606	<0.0001	Yes
Post-OS Pc DNA vs. Pc Nuclei	22	0.4636	0.0388 to 0.7465	0.0298	Yes
Pre-OS Pc DNA vs. post-OS Pc DNA	21^*^	0.3707	-0.0863 to 0.6988	0.0980	No
Pre-OS Pc DNA vs Pc Nuclei	21	0.4123	-0.0374 to 0.7232	0.0633	No
Pre-OS Pc DNA vs. LH Pc DNA	21	0.2939	-0.1712 to 0.6519	0.1960	No

^a^21 data points were included in these analyses because 2 rats from the 10-wk group died and 1 preimmunosuppression oral swab sample in the 7-wk group was lost in processing. ^b^Pre-OS Pc DNA, *P. carinii* –specific DNA from oral swabs taken prior to immunosuppression; post-OS Pc DNA, *P. carinii*–specific DNA from oral swabs taken at the time of euthanasia; LH Pc DNA, *P. carinii*–specific DNA from lung homogenates of rats at the 3 different time points; log Pc Nuclei, *P. carinii* organism number assessed by microscopic enumeration.

## Conclusions

The combination of antemortem oral swab sampling and real-time PCR amplification and quantification reported here should be useful for the study of the *Pneumocystis* infections in other experimental models and provides a rationale for similar studies to be conducted in the clinical setting. Real-time PCR previously has been shown to be useful for quantitation of the level of infection in the lungs of infected rats and mice, but the studies were performed on postmortem samples or purified organisms (*8*,*9*) *P. jiroveci* DNA levels from oral washes, induced sputa, and bronchoalveolar lavage fluids from humans have been quantified by using various real-time PCR techniques (*10*–*13*) as well, but the findings were used for diagnosis, detection, or quantification and did not obtain samples from individual hosts over time. In our study, the levels of *P. carinii* DNA in the oral cavities of the rats were measured temporally and shown to correlate with the numbers of organisms in the lungs, establishing the oral swab real-time PCR technique as a surrogate means of following the progress of the infection. Successful application of this method to the human infection would enhance epidemiologic studies, permit sensitive and rapid assessment of therapeutic response, and allow basic biologic questions of carriage length and potential reservoirs to be addressed.
